# Real‐World Evidence That Non‐Smokers With High PD‐L1 Non‐Squamous NSCLC Have Poorer Outcomes With Immune Checkpoint Inhibitors

**DOI:** 10.1111/1759-7714.70167

**Published:** 2025-09-17

**Authors:** Yu‐Chu Kuo, Wen‐Chien Cheng, Hsu‐Yuan Chen, Chun‐Ru Chien, Chih‐Yen Tu, Hung‐Jen Chen

**Affiliations:** ^1^ Division of Pulmonary and Critical Care Medicine Taichung Taiwan; ^2^ China Medical University Hospital Taichung Taiwan; ^3^ School of Medicine, College of Medicine China Medical University Taichung Taiwan; ^4^ Department of Radiation Oncology China Medical University Hospital Taichung Taiwan

**Keywords:** chemoimmunotherapy, immune checkpoint inhibitors, non‐small cell lung cancer, non‐squamous histology, PD‐L1, smoking

## Abstract

**Background:**

Immune checkpoint inhibitors (ICIs) improve outcomes in non‐small cell lung cancer (NSCLC) with high PD‐L1 expression, but biomarkers beyond PD‐L1 are limited. Smoking‐related immune activation may enhance ICI efficacy, yet evidence in non‐squamous NSCLC, especially among non‐smokers, is sparse.

**Methods:**

We retrospectively analyzed 74 patients with Stage IIIB–IV non‐squamous NSCLC, PD‐L1 ≥ 50%, and no *EGFR/ALK/ROS1* mutations, treated at a tertiary center in Taiwan (2017–2023). Patients were stratified by smoking status. Treatment responses, progression‐free survival (PFS), and overall survival (OS) were evaluated using RECIST v1.1, Kaplan–Meier, and Cox regression.

**Results:**

Among 74 patients, 54 (72.9%) were smokers and 20 (27.1%) were non‐smokers. Compared with non‐smokers, smokers had a higher partial response rate (66.7% vs. 25.0%, *p* = 0.001), longer median PFS (12.8 vs. 1.4 months, *p* = 0.001), and improved OS (47.1 vs. 10.0 months, *p* = 0.011). In the non‐smoker subgroup, chemoimmunotherapy significantly prolonged PFS compared with ICI monotherapy (not reached vs. 1.4 months, *p* = 0.034). In multivariate analysis, smoking independently predicted better PFS (HR = 0.234, *p* = 0.001) and OS (HR = 0.229, *p* = 0.011).

**Conclusion:**

Non‐smokers with PD‐L1‐high non‐squamous NSCLC showed significantly poorer outcomes with ICI monotherapy. Chemoimmunotherapy may be preferred in this group. Smoking history may provide a simple and clinically relevant stratification factor when considering ICI‐based treatment.

## Graphical abstract

Non‐smokers with PD‐L1‐high non‐squamous NSCLC showed significantly poorer outcomes with ICI therapy. Chemoimmunotherapy may be more effective in this subgroup.

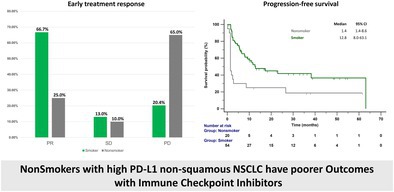

**Abbreviations**
ALK: anaplastic lymphoma kinase
CI: confidence interval
CR: complete response
CT: computed tomography
EGFR: epidermal growth factor receptor
HR: hazard ratio
ICI: immune checkpoint inhibitor
irAE: immune‐related adverse event
NSCLC: non‐small cell lung cancer
ORR: objective response rate
OS: overall survival
PD: progressive disease
PD‐L1: programmed death‐ligand 1
PFS: progression‐free survival
PR: partial response
RECIST: response evaluation criteria in solid tumors
SD: stable disease
TMB: tumor mutational burden
TPS: tumor proportion score

## 1 Introduction

Lung cancer remains the most common cause of cancer‐related mortality worldwide, accounting for approximately 18% of all cancer deaths [1]. The advent of immune checkpoint inhibitors (ICIs), particularly programmed cell death protein 1 (PD‐1) and programmed death‐ligand 1 (PD‐L1) inhibitors such as pembrolizumab, has substantially improved survival outcomes in advanced non‐small cell lung cancer (NSCLC), especially in patients whose tumors express high levels of PD‐L1 (tumor proportion score (TPS) ≥ 50%) and who lack driver mutations in *EGFR* or *ALK* [2, 3]. Despite these advances, response rates to ICI monotherapy in this population remain limited—typically around 45%–50%—suggesting that PD‐L1 expression alone is insufficient as a predictive biomarker [3].

Tobacco smoking, the primary risk factor for lung cancer, has been associated with a higher tumor mutational burden (TMB), neoantigen load, and immune cell infiltration, all of which may enhance response to ICIs [4, 5]. Previous studies have suggested improved outcomes in smokers treated with ICIs, but statistical significance was often lacking [3, 6, 7], possibly due to mixed histologic populations and small sample sizes. Moreover, major prognostic models such as LIPS‐3 and other early progression scores have not identified smoking as an independent prognostic factor for patients with high PD‐L1 expression treated with pembrolizumab [8, 9].

Importantly, most existing studies group squamous and non‐squamous NSCLC together despite significant differences in biology, immune environment, and treatment response [10, 11]. Non‐squamous histology, especially adenocarcinoma, predominates in East Asian populations and in never‐smokers, who often exhibit unique molecular profiles such as *EGFR, ALK, ROS1, HER2*, or *RET* alterations [12]. Emerging evidence suggests that non‐smokers may derive less benefit from ICI therapy despite high PD‐L1 expression. However, no prior study has focused exclusively on this subgroup to validate this observation and the optimal use of monotherapy versus combination chemo‐immunotherapy.

This retrospective real‐world study aimed to compare the treatment responses, progression‐free survival (PFS), and overall survival (OS) between smokers and non‐smokers in this specific NSCLC subgroup, and to explore whether treatment strategies should be tailored according to smoking history.

## 2 Materials and Methods

### 2.1 Study Population

This retrospective study analyzed a prospective registry of patients with stage IIIB–IV non‐squamous NSCLC, as classified by the American Joint Committee on Cancer Eighth Edition [13], at China Medical University Hospital, a major tertiary referral center in Taiwan, between August 2017 and July 2023. Patients were excluded if they harbored *EGFR, ALK*, or *ROS1* mutations or had missing critical clinical data.

PD‐L1 expression was assessed using the TPS, defined as the percentage of tumor cells exhibiting membranous staining, based on immunohistochemical assays (Dako 22C3 or Ventana SP263). High PD‐L1 expression was defined as TPS ≥ 50%. Smoking status was classified as smokers (≥ 100 cigarettes in a lifetime) or non‐smokers, following standard definitions [14]. Additionally, smoking exposure was summarized by the Brinkman Index (BI, cigarettes/day × years) for exploratory analyses, stratified as BI < 200 [15] and BI ≥ 200.

Baseline clinical characteristics, obtained from electronic medical records, included age, sex, tumor histology, tumor‐node‐metastasis stage at diagnosis, sites of distant metastasis, initial and subsequent treatments, treatment modality (ICI monotherapy or chemo‐immunotherapy), treatment duration, and occurrence of immune‐related adverse events (irAEs). The study was approved by the Institutional Review Board of China Medical University Hospital (approval number: CMUH113‐REC1‐021). The requirement for informed consent was waived due to the retrospective study design. The authors had access to de‐identified patient information only and did not retain or analyze any personally identifiable data during or after data collection.

### 2.2 Treatment and Safety Evaluation

Treatment strategies, including ICIs and/or chemotherapy, were determined at the discretion of the treating physicians. Following treatment initiation, patients underwent chest radiography every 2–4 weeks and chest computed tomography (CT) every 12–16 weeks. Additional imaging (e.g., positron emission tomography, bone scans, brain CT, or magnetic resonance imaging) was performed as clinically indicated.

Treatment response was evaluated using the Response Evaluation Criteria in Solid Tumors (RECIST), version 1.1 [16], and classified as complete response (CR), partial response (PR), stable disease (SD), or progressive disease (PD). PFS was defined as the interval from ICI initiation to documented disease progression, death, or last follow‐up. OS was defined as the time from initiation of ICI therapy to death or last follow‐up (March 31, 2024).

### 2.3 Statistical Analysis

Categorical variables were summarized as counts (percentages) and compared using the chi‐square test. Ordinal variables were analyzed with the Kruskal–Wallis test. Kaplan–Meier survival curves were used to estimate PFS and OS.

Variables with statistical significance in univariate analysis, along with clinically relevant covariates, were included in a multivariate Cox proportional hazards model to control for potential confounding factors. Results were expressed as hazard ratios (HRs) with 95% confidence intervals (CIs). Logistic regression analysis was additionally performed to evaluate independent predictors of objective response. A two‐tailed *p* value < 0.05 was considered statistically significant. All statistical analyses were conducted using MedCalc for Windows (version 18.10; MedCalc Software, Ostend, Belgium).

## 3 Results

### 3.1 Patient Characteristics

**FIGURE 1:**
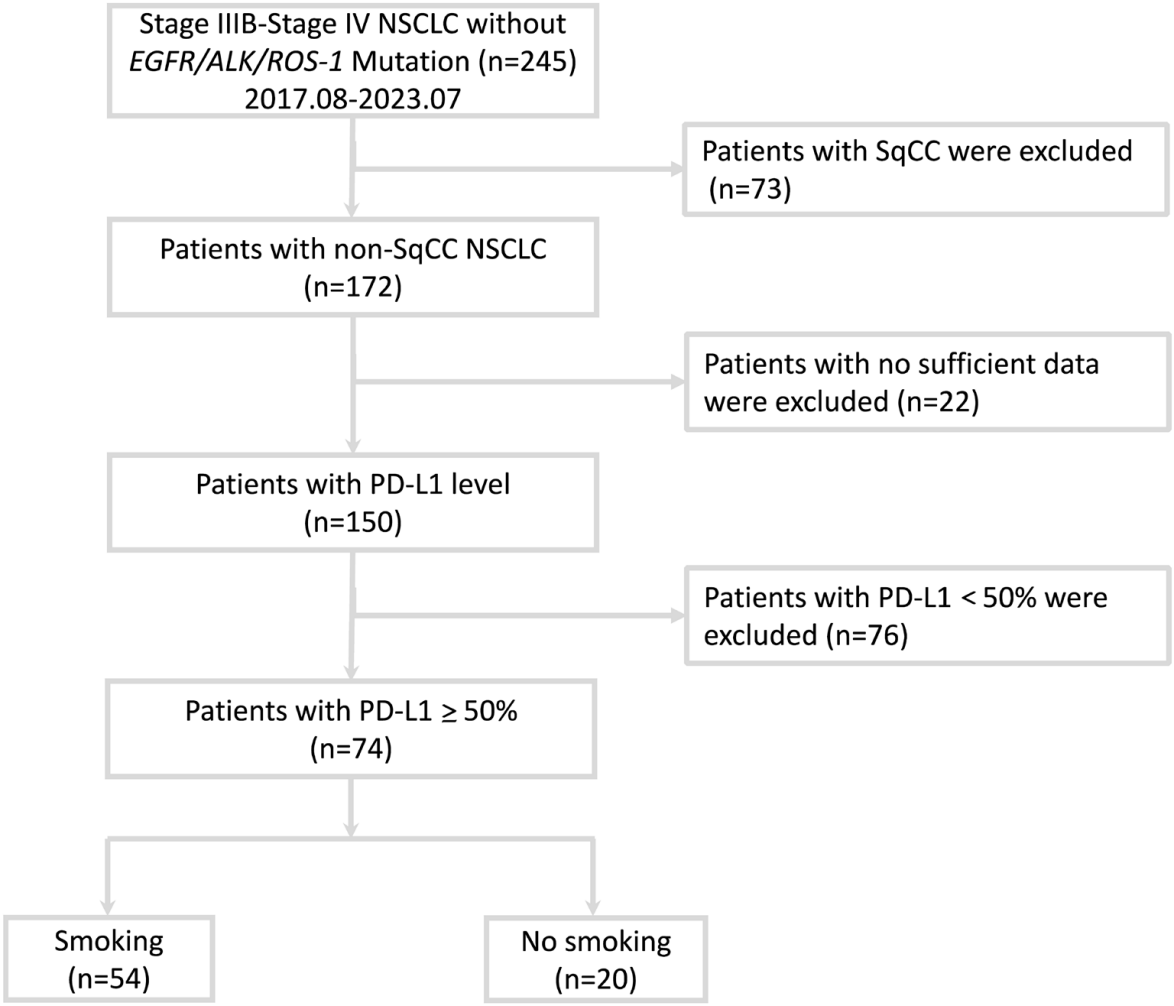
Flowchart of patient enrollment. Abbreviations: ALK, anaplastic lymphoma kinase; EGFR, epidermal growth factor receptor; NSCLC, non‐small cell lung cancer; PD‐L1, programmed death‐ligand 1.

Between August 2017 and July 2023, a total of 172 patients were diagnosed with stage IIIB–IV non‐squamous NSCLC without *EGFR, ALK*, or *ROS1* mutations. After excluding 22 patients with incomplete data, 76 with PD‐L1 expression < 50%, 74 eligible patients were included in the final analysis (Figure 1). Among them, 54 (72.9%) were smokers and 20 (27.1%) were non‐smokers. The median follow‐up duration was 30.2 months (95% CI: 16.5–36.5 months).

Baseline clinical characteristics are presented in Table 1. A significantly greater proportion of smokers was male compared to non‐smokers (92.6% vs. 35.0%, *p* < 0.001). Bone metastasis was more frequently observed in non‐smokers than smokers (45.0% vs. 27.8%), although this difference did not reach statistical significance (*p* = 0.162). A higher proportion of smokers received combination therapy with ICIs and chemotherapy compared to non‐smokers (44.4% vs. 10.0%, *p* = 0.006). No significant differences were observed between the groups in terms of age, histology, cancer stage, or other metastatic sites. The incidence of irAEs was similar between smokers (11.1%) and non‐smokers (15.0%).

**TABLE 1:** Baseline clinical characteristics of smokers and non‐smokers with non‐squamous NSCLC (PD‐L1 ≥ 50%).

Variable	Smoking (*n* = 54)	Non‐smoking (*n* = 20)	*p*
Age ≥ 65 years—no. (%)	29 (53.7)	13 (65.0)	0.386
Male—no. (%)	50 (92.6)	7 (35.0)	< 0.001
ECOG PS—no. (%)			0.637
0	20 (55.6)	10 (58.8)	
1	14 (38.9)	5 (29.4)	
2	2 (5.6)	2 (11.8)	
Histology—no. (%)			0.437
Adenocarcinoma	48 (88.9)	17 (85.0)	
Others	6 (11.1)	3 (15.0)	
Stage—no. (%)			0.863
IIIB/IIIC	9 (16.7)	3 (15.0)	
IV	45 (83.3)	17 (85.0)	
Metastasis—no. (%)			
Lung	4 (7.4)	3 (15.0)	0.325
Pleural	9 (16.7)	2 (10.0)	0.477
Liver	5 (9.3)	4 (20.0)	0.212
Bone	15 (27.8)	9 (45.0)	0.162
CNS	15 (27.8)	7 (35.0)	0.548
Adrenal	6 (11.1)	2 (10.0)	0.892
Immunotherapy—no. (%)			0.220
Pembrolizumab	49 (90.7)	18 (90.0)	
Others	5 (9.3)	2 (10.0)	
ICI first‐line—no. (%)	39 (72.2)	12 (60.0)	0.316
ICIs + CT—no. (%)	24 (44.4)	2 (10.0)	0.006
irAE—no. (%)	6 (11.1)	3 (15.0)	0.651
Response—no. (%)			0.001
Partial response	36 (66.7)	5 (25.0)	
Stable disease	7 (13.0)	2 (10.0)	
Progressive disease	11 (20.4)	13 (65.0)	

Abbreviations: CNS, central nervous system; CT, chemotherapy; ECOG PS, eastern cooperative oncology group performance status; ICI, immune checkpoint inhibitor; irAE, immune‐related adverse event; PD, progressive disease; PR, partial response; SD, stable disease.

### 3.2 Treatment Response and Survival Outcomes

**FIGURE 2:**
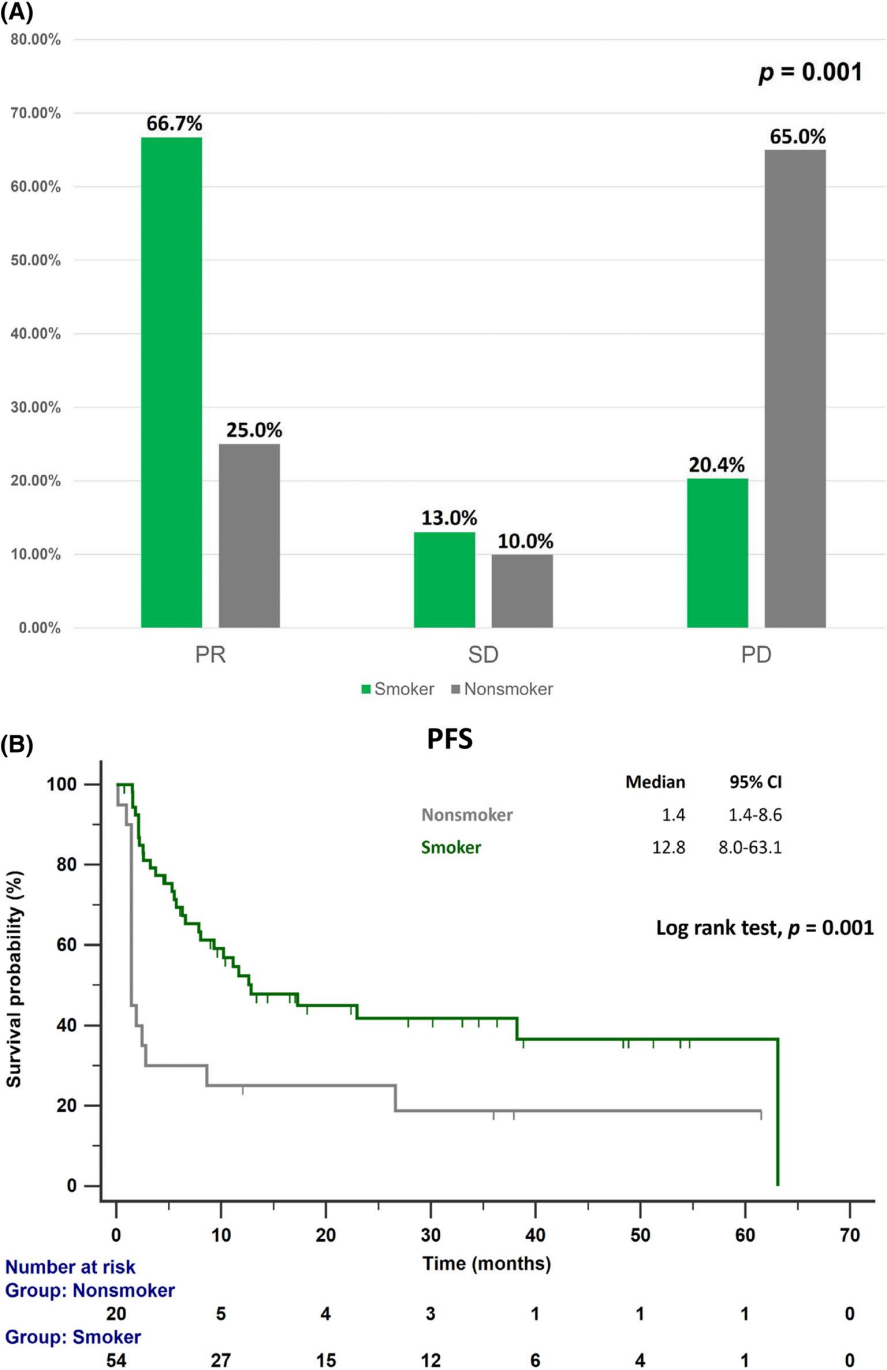
(A) Comparison of early treatment response to ICIs within 3 months between smokers and non‐smokers. (B) Kaplan–Meier PFS curves for smokers and non‐smokers with non‐squamous NSCLC and PD‐L1 ≥ 50% treated with ICIs. Abbreviations: ICIs, immune checkpoint inhibitors; NSCLC, non‐small cell lung cancer; PD, progressive disease; PFS, progression‐free survival; PR, partial response; SD, stable disease.

Treatment responses, evaluated according to RECIST version 1.1 [16], differed significantly between the two groups (*p* = 0.001; Figure 2A). Among smokers, 66.7% achieved a PR, 13.0% had SD, and 20.4% experienced PD. In contrast, non‐smokers exhibited a PR rate of 25.0%, an SD rate of 10.0%, and a PD rate of 65.0%. Median PFS was 12.8 months for smokers and 1.4 months for non‐smokers (*p* = 0.001; Figure 2B).

**FIGURE 3:**
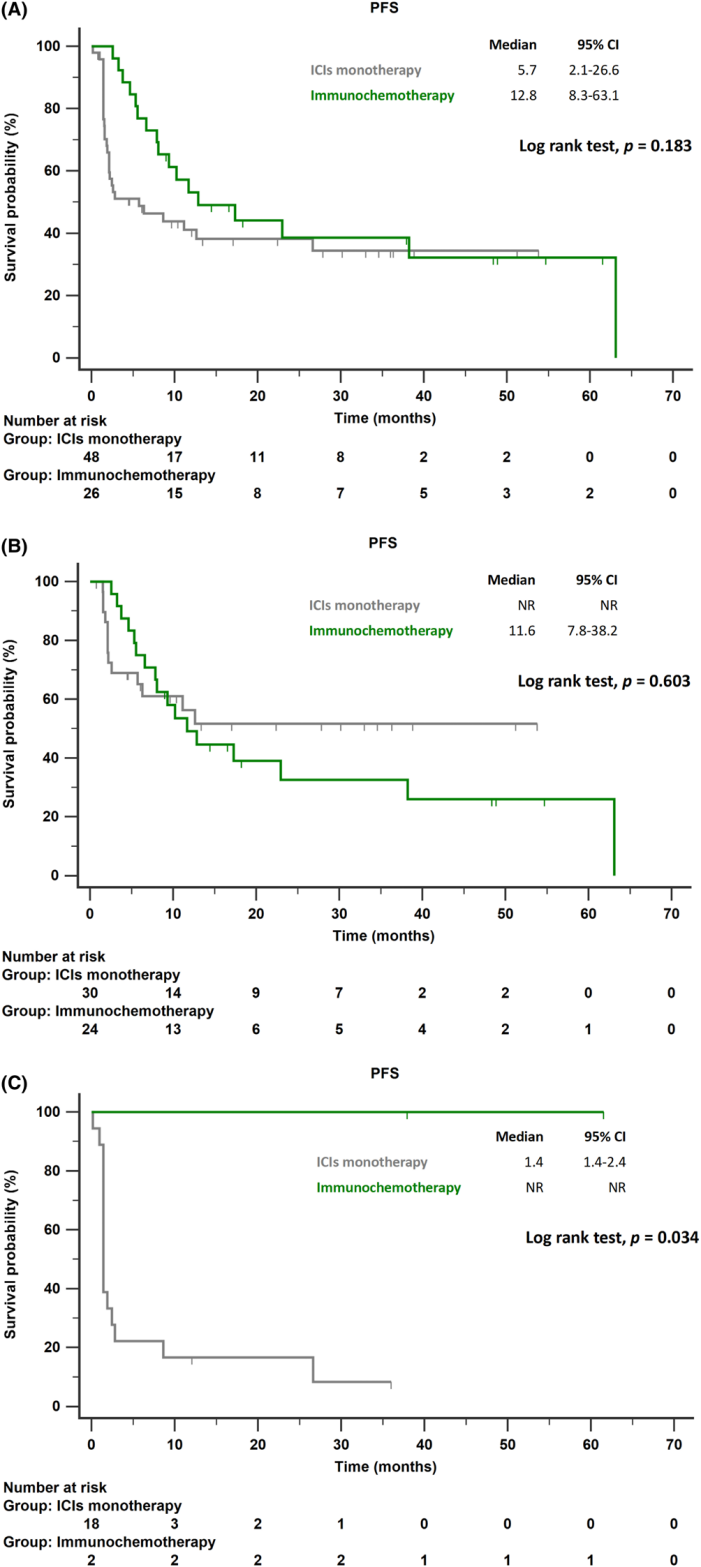
PFS stratified by treatment type (ICI monotherapy vs. immunochemotherapy) in patients with non‐squamous NSCLC and PD‐L1 ≥ 50%. (A) All patients. (B) Smokers. (C) Non‐smokers. Abbreviations: ICI, immune checkpoint inhibitor; NSCLC, non‐small cell lung cancer; PFS, progression‐free survival.

In the entire cohort, patients treated with ICI monotherapy had a median PFS of 5.7 months (95% CI: 2.1–26.6), while those receiving chemoimmunotherapy had a median PFS of 12.8 months (95% CI: 8.3–63.1; *p* = 0.183; Figure 3A). Among smokers, the median PFS was not reached in the ICI monotherapy group and was 11.6 months (95% CI: 7.8–38.2) in the chemoimmunotherapy group (*p* = 0.603; Figure 3B). Among non‐smokers, median PFS was 1.4 months (95% CI: 1.4–2.4) with ICI monotherapy and was not reached with combination therapy (*p* = 0.034; Figure 3C).

**FIGURE 4:**
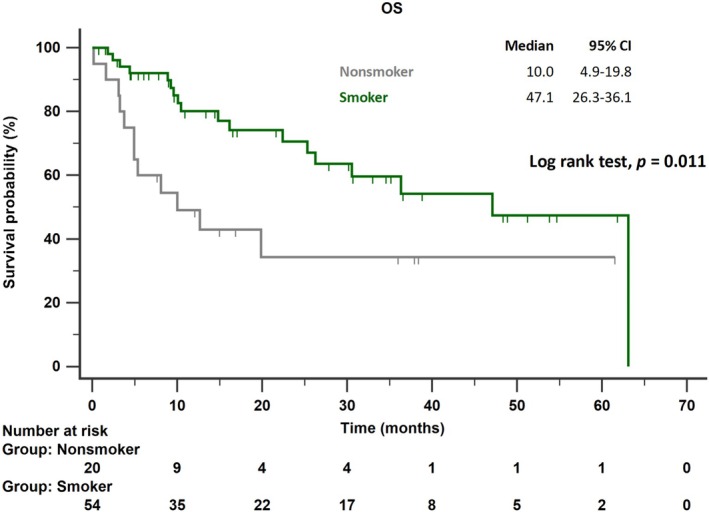
Kaplan–Meier curves for OS in smokers and non‐smokers with advanced non‐squamous NSCLC and PD‐L1 expression ≥ 50% treated with ICIs. Abbreviations: ICIs, immune checkpoint inhibitors; NSCLC, non‐small cell lung cancer; OS, overall survival.

Median OS was 47.1 months (95% CI: 26.3–36.1) in smokers, whereas it was 10.0 months (95% CI: 4.9–19.8) in non‐smokers (*p* = 0.011; Figure 4).

Because more smokers received chemoimmunotherapy, we constructed multivariate models including age, sex, ECOG PS, disease stage, treatment regimen, bone metastasis, and smoking. In logistic regression for objective response (Table S1), smoking remained an independent predictor (odds ratio 11.62, 95% CI 1.71–78.9, *p* = 0.012). In Cox regression models for survival (Tables 2 and 3), smoking also independently predicted improved outcomes for both PFS (HR = 0.234, 95% CI: 0.09–0.56; *p* = 0.001) and OS (HR = 0.229, 95% CI: 0.07–0.71; *p* = 0.011), whereas male sex and bone metastasis were associated with poorer prognosis.

**TABLE 2:** Independent predictors of PFS in patients treated with ICIs.

	Univariate			Multivariate		
	HR	95% CI	*p*	HR	95% CI	*p*
Age > 65 years	0.986	0.55–1.77	0.963	0.863	0.47–1.58	0.863
Sex, male	0.831	0.42–1.64	0.594	2.421	0.93–6.28	0.069
ECOG PS ≥ 2	0.634	0.19–2.05	0.447	0.621	0.18–2.11	0.446
Stage IV versus III	1.748	0.73–4.17	0.208	1.338	0.51–3.53	0.555
Smoking	0.378	0.21–0.71	0.002	0.234	0.09–0.56	0.001
ICIs + CT	0.663	0.36–1.23	0.191	0.884	0.43–1.81	0.735
Bone M	2.056	1.15–3.79	0.021	2.028	1.01–4.05	0.045

Abbreviations: CI, confidence interval; CT, chemotherapy; ECOG PS, eastern cooperative oncology group performance status; HR, hazard ratio; ICIs, immune checkpoint inhibitors; M, metastasis; PFS, progression‐free survival.

**TABLE 3:** Independent predictors of OS in patients treated with ICIs.

	Univariate			Multivariate		
	HR	95% CI	*p*	HR	95% CI	*p*
Age > 65 years	1.545	0.72–3.28	0.257	1.267	0.55–2.91	0.557
Sex, male	0.885	0.39–2.01	0.885	3.794	1.21–11.9	0.022
ECOG PS ≥ 2	0.711	0.17–2.99	0.642	0.722	0.16–3.24	0.671
Stage IV versus III	7.457	1.01–54.9	0.048	4.409	0.55–34.9	0.161
Smoking	0.395	0.18–0.83	0.015	0.229	0.07–0.71	0.011
ICIs + CT	0.604	0.27–1.31	0.204	0.809	0.31–2.11	0.666
Bone M	4.167	1.96–8.84	< 0.001	3.691	1.64–8.31	0.002

Abbreviations: CI, confidence interval; CT, chemotherapy; ECOG PS, eastern cooperative oncology group performance status; HR, hazard ratio; ICIs, immune checkpoint inhibitors; M, metastasis; PFS, progression‐free survival.

In an exploratory analysis stratified by Brinkman Index (Table S2), patients with BI ≥ 200 had higher objective response rates (ORRs) and longer PFS/OS compared with those with BI < 200, with non‐smokers showing the poorest outcomes. Given the very small BI < 200 subgroup (*n* = 3), these results are descriptive.

## 4 Discussion

To our knowledge, this is the first real‐world study specifically evaluating the impact of smoking history on ICI treatment outcomes in patients with advanced non‐squamous NSCLC, high PD‐L1 expression (≥ 50%), and no *EGFR*/*ALK*/*ROS1* alterations. Our findings demonstrate that smokers had significantly better treatment responses—including PR rate (66.7% vs. 25.0%)—as well as longer PFS (12.8 vs. 1.4 months) and OS (47.1 vs. 10.0 months) compared to non‐smokers. Notably, among non‐smokers, those receiving chemoimmunotherapy showed a significant improvement in PFS compared to those receiving ICI monotherapy, despite the limited sample size.

Our results are consistent with prior observations that smokers may benefit more from ICIs. The KEYNOTE‐024 and IMpower110 trials showed improved survival with ICI monotherapy in patients with PD‐L1 ≥ 50%, with numerically greater benefits among smokers, though statistical significance was not reached—likely due to the low prevalence of non‐smokers in those trials (≤ 25%) [17, 18]. Gainor et al. reported lower response durability in never‐smokers compared to light or heavy smokers, despite similar ORRs across groups [6]. In contrast, larger meta‐analyses have demonstrated statistically significant PFS benefits in smokers, but not in non‐smokers, with PD‐L1 ≥ 50% NSCLC receiving ICI therapy [7]. Popat et al. also found a clear survival advantage for smokers in a real‐world cohort receiving first‐line pembrolizumab [19].

Unlike earlier studies that mixed squamous and non‐squamous histologies or included *EGFR*‐mutated patients [17, 18], our study specifically excluded those confounding factors, allowing for a clearer signal within a biologically coherent subgroup. This may explain why statistically significant differences in OS and PFS were still observed, despite the small sample size.

The observed outcome differences likely stem from biological disparities. Smoking is associated with elevated TMB, increased CD8+ T‐cell infiltration, and enrichment of *KRAS* mutations, all of which contribute to better ICI responses [4, 5, 20]. In contrast, non‐smokers may harbor alterations such as *STK11* or *KEAP1* mutations that impair response [21, 22], or *HER2/RET* fusions not covered under standard *EGFR/ALK* testing. Moreover, in Taiwan, PCR‐based *EGFR* testing may yield false negatives compared to next‐generation sequencing platforms [23, 24], potentially misclassifying some patients. This may partially explain the poor response in non‐smokers with high PD‐L1 levels. Additionally, the tumor immune microenvironment in non‐smokers may be less inflamed, with lower T‐cell infiltration and antigenicity, further reducing ICI efficacy [12].

To further explore the dose–response relationship, we performed a Brinkman Index–based analysis, which demonstrated a graded pattern (BI ≥ 200 > BI < 200 > non‐smokers). Although underpowered due to the very small BI < 200 subgroup, this finding supports the biological rationale that heavier tobacco exposure is associated with more inflamed tumor microenvironments and improved ICI efficacy.

In our analysis, male sex was also associated with worse outcomes (Table 3). Nevertheless, despite the majority of smokers being men, smokers still demonstrated superior ICI efficacy compared with non‐smokers.

Clinically, our findings support a stratified treatment approach. While ICI monotherapy remains a standard for PD‐L1 ≥ 50% NSCLC [3], our data suggest that non‐smokers may benefit more from combination chemoimmunotherapy. This aligns with network meta‐analyses showing improved PFS and ORR with combination regimens in high PD‐L1 expressers [25, 26].

Furthermore, bone metastasis independently predicted worse outcomes in our multivariate models, consistent with prior studies [27]. These variables should be considered in future prognostic models alongside smoking status.

Our study has limitations. The retrospective design and single‐center setting may introduce selection bias. The relatively small sample size, especially in the non‐smoker subgroup (only two patients received chemoimmunotherapy), limited statistical power for subgroup and multivariate analyses. These findings should therefore be interpreted with caution and validated in larger, prospective cohorts. Additionally, molecular alterations beyond *EGFR/ALK/ROS1* were not captured due to limitations in insurance coverage, precluding analysis of *KRAS, STK11, KEAP1, HER2*, or *RET* status. Finally, we could not validate prior prognostic scoring systems such as LIPS‐3 [8] or early progression models [9] due to the limited sample size.

Despite these limitations, our findings highlight smoking history as a clinically relevant predictor of ICI efficacy in this specific NSCLC subgroup. They also suggest that non‐smokers with PD‐L1 ≥ 50% should be carefully evaluated for combination therapy rather than relying solely on monotherapy.

## 5 Conclusion

In conclusion, this real‐world cohort study demonstrates that among patients with advanced non‐squamous NSCLC with PD‐L1 ≥ 50% and no targetable mutations, smoking status is a significant predictor of ICI treatment outcomes. Smokers showed superior response, PFS, and OS compared to non‐smokers. Non‐smokers may derive greater benefit from chemoimmunotherapy than ICI monotherapy. These findings underscore the importance of incorporating smoking history and biological context into treatment decision‐making and support further investigation in larger, prospective, biomarker‐integrated studies.

## Author Contributions

Y.‐C.K., W.‐C.C., and H.‐J.C. did the study design. Y.‐C.K., W.‐C.C., H.‐Y.C., and H.‐J.C. contributed to patient enrollment and data collection. Y.‐C.K., W.‐C.C., C.‐R.C., and H.‐J.C. analyzed the data. Y.‐C.K. and W.‐C.C. drafted the manuscript. W.‐C.C. and H.‐J.C. have seen and can confirm the authenticity of the raw data. Y.‐C.K., W.‐C.C., H.‐Y.C., and H.‐J.C. approved the final version of the manuscript to be published.

## Ethics Statement

This retrospective study was approved by the Institutional Review Board of China Medical University Hospital (approval no. CMUH113‐REC1‐021) and was conducted in accordance with the ethical principles of the Declaration of Helsinki.

## Consent

Informed consent was waived due to the noninterventional and retrospective design.

## Conflicts of Interest

The authors declare no conflicts of interest.

## Supporting information


**Table S1:** Logistic regression for predictors of objective response after ICI treatment.

**Table S2:** Outcomes stratified by Brinkman Index (BI).

## Data Availability

The datasets generated and analyzed during the current study are not publicly available due to restrictions imposed by the China Medical University Hospital Institutional Review Board. However, de‐identified data can be made available from the corresponding author upon reasonable request.
