# Supplementary material for: Real‐World Evidence That Non‐Smokers With High PD‐L1 Non‐Squamous NSCLC Have Poorer Outcomes With Immune Checkpoint Inhibitors
Source: Thorac Cancer. 2025 Sep 17;16(18):e70167. doi: 10.1111/1759-7714.70167 (PMC12443809; doi:10.1111/1759-7714.70167)
Supplement: Supplementary file 1 — Table S1: Logistic regression for predictors of objective response after ICI treatment. [file TCA-16-e70167-s001.docx]

## Supplementary Table S1. Logistic regression for predictors of objective response after ICI treatment

|  | Univariate | | |  | Multivariate | | |
| --- | --- | --- | --- | --- | --- | --- | --- |
|  | OR | 95% CI | *p*-value |  | OR | 95% CI | *p*-value |
| Age > 65 years | 1.176 | 0.46–2.96 | 0.731 |  | 1.713 | 0.57–5.12 | 0.336 |
| Sex, male | 2.112 | 0.71–6.35 | 0.183 |  | 0.338 | 0.05–2.24 | 0.261 |
| ECOG PS ≥2 | 4.444 | 0.49–40.1 | 0.183 |  | 5.055 | 0.41–63.6 | 0.209 |
| Stage IV vs. III | 1.296 | 0.38–4.47 | 0.681 |  | 1.734 | 0.38–7.81 | 0.472 |
| Smoking | 6.001 | 1.88–19.1 | 0.003 |  | 11.62 | 1.71–78.9 | 0.012 |
| ICIs + CT | 3.207 | 1.14–9.04 | 0.022 |  | 1.653 | 0.49–5.51 | 0.412 |
| Bone M | 0.337 | 0.12–0.92 | 0.034 |  | 0.325 | 0.09–1.08 | 0.068 |

Abbreviations: ECOG PS, Eastern Cooperative Oncology Group Performance Status; CT, chemotherapy; ICIs, immune checkpoint inhibitors; OR, odds ratio; CI, confidence interval; M, metastasis

This table shows univariate and multivariate logistic regression analyses for objective response in patients with advanced non-squamous NSCLC (PD-L1 ≥50%) treated with ICIs, adjusting for key clinical and treatment-related factors. Smoking remained an independent predictor of objective response after adjustment..
