# Supplementary material for: Real‐World Evidence That Non‐Smokers With High PD‐L1 Non‐Squamous NSCLC Have Poorer Outcomes With Immune Checkpoint Inhibitors
Source: Thorac Cancer. 2025 Sep 17;16(18):e70167. doi: 10.1111/1759-7714.70167 (PMC12443809; doi:10.1111/1759-7714.70167)
Supplement: Supplementary file 2 — Table S2: Outcomes stratified by Brinkman Index (BI). [file TCA-16-e70167-s002.docx]

## Supplementary Table S2. Outcomes stratified by Brinkman Index (BI)

| Group | n | ORR (%) | Median PFS  months (95% CI) | Median OS  months (95% CI) |
| --- | --- | --- | --- | --- |
| BI ≥200  (heavier exposure) | 51 | 70.6% | 17.2 (8.1–63.1) | 47.1 (30.5–63.1) |
| BI <200  (lighter exposure) | 3 | 0% | 2.2 (1.5–10.2) | 26.3 (NR) |
| Non-smokers | 20 | 25.0% | 1.4 (1.4–8.6) | 10.0 (4.9–19.8) |

Abbreviations: ORR, objective response rate; PFS, progression-free survival; OS, overall survival; NR, not reached. Statistical testing was not performed due to the very small number of patients in the BI <200 subgroup (n=3).
